# Serum and Tissue Periostin Expression in Unilateral Benign Lesions of the Nose and the Paranasal Sinuses

**DOI:** 10.3390/jpm14121156

**Published:** 2024-12-19

**Authors:** Gerasimos Danielides, Alkmini Gatsounia, George Kyriakopoulos, Constantinos Stathopoulos, Stephanos Naxakis, Spyridon Lygeros

**Affiliations:** 1 Department of Otorhinolaryngology, Medical School, University of Patras, GR 26504 Patras, Greece; up1096481@ac.upatras.gr (G.D.); snaxakis@upatras.gr (S.N.); slygeros@upatras.gr (S.L.); 2 Department of Biochemistry, Medical School, University of Patras, GR 26504 Patras, Greece; g.kyriakopoulos@ac.upatras.gr (G.K.); cstath@upatras.gr (C.S.)

**Keywords:** periostin, biomarker, nasal mucosa, inverted papilloma, mucocele

## Abstract

**Background/Objectives**: Periostin’s role in the pathogenesis of inflammatory diseases, particularly in the nose and paranasal sinuses, is an area of growing interest. This study aims to evaluate the expression of periostin in mucoceles, inverted papillomas, choanopolyps and retention cysts. **Methods**: Tissue samples collected during functional endoscopic sinus surgery (FESS) were analyzed for POSTN gene mRNA expression using qPCR. Periostin protein levels were measured via ELISA and Western Blot. Serum periostin levels were also assessed through ELISA in both patients (*n* = 54) and controls (*n* = 12). **Results**: A total of 66 participants were recruited, including 18 with inverted papillomas, 10 with mucoceles, 10 with choanopolyps, 16 with retention cysts and 12 controls. There were no significant alternations between tissue and serum samples of inverted papilloma compared to the control group. Choanopolyp tissues exhibited elevated POSTN protein expression, though POSTN mRNA and serum levels remained unchanged. In mucoceles, periostin levels were significantly elevated in both tissues and serum. Retention cyst tissues demonstrated an increase in POSTN mRNA and protein expression, whereas serum periostin levels remained consistent with those observed in the control group. **Conclusions**: The findings suggest that periostin may play a role in the pathophysiology of benign non-neoplastic lesions of the nose and paranasal sinuses such as mucoceles, retention cysts and choanopolyps, highlighting a need for more investigation in this subject.

## 1. Introduction

Periostin (POSTN), originally isolated from an osteoblast cell line, is a 90 kDa N-glycoprotein involved in bone development that has garnered significant attention for its pivotal roles in tissue repair, remodeling, and immune responses [1]. As an extracellular matrix (ECM) protein, periostin is induced by IL-4 and IL-13 in airway epithelial cells, promoting collagen fibrillation and cross-linking [2]. This matricellular signaling protein regulates ECM–cell interactions and thus controls fibrotic and non-fibrotic pathways in a variety of organs and tissues [3]. It is involved in fibrosis, collagen deposition and fibrotic remodeling and has been implicated in several chronic inflammatory diseases and a myriad of biological processes, extending its influence beyond mere structural support.

In rhinology, periostin has been studied extensively for its role in Th2-mediated inflammation, particularly in conditions like chronic rhinosinusitis (CRS) and its subtypes, including eosinophilic chronic rhinosinusitis (ECRS) and chronic rhinosinusitis with nasal polyps (CRSwNP) [4]. These conditions are characterized by persistent inflammation of the nasal and paranasal sinus mucosa and present a substantial public health burden, affecting a significant portion of the population and impinging on quality of life. The role of POSTN in tissue remodeling and fibrosis is particularly relevant in the context of the chronic inflammatory environment and tissue remodeling observed in CRS and ECRS, which have been key areas of scientific investigation in recent years [5]. The upregulation of POSTN in these conditions points to its potential involvement in the pathophysiological processes, possibly mediating between inflammatory responses and tissue remodeling outcomes such as nasal polyp formation.

While periostin’s involvement in Th-2 inflammation has been widely studied, its functions extend beyond this context. As an extracellular matrix protein, periostin is implicated in key roles in pathways related to autoimmune diseases, particularly those affecting the skin and lungs, as well as in cancers [6]. Additionally, periostin is hypothesized to play a pivotal role in cancer growth and progression [7]. Furthermore, the interaction of POSTN with different cell types and signaling pathways indicates the existence of a complex regulatory network that influences disease progression and severity [8,9]. This has significant potential for use as a biomarker [10].

Despite advances in understanding periostin’s role in inflammatory diseases, its function in benign nasal and paranasal lesions, such as inverted papillomas, mucoceles, choanopolyps, and retention cysts, remains unexplored. These lesions can mimic CRSwNP due to their polypoid appearance in nasoendoscopy, presenting diagnostic challenges. Investigating periostin’s expression in these conditions could provide insights into its role in tissue remodeling and aid in differential diagnosis. Furthermore, periostin’s association with fibrosis and ECM remodeling raises the possibility of its involvement in the development of these lesions, potentially offering novel therapeutic and diagnostic avenues.

This study aims to evaluate periostin expression in benign nasal lesions, emphasizing its potential as a biomarker and therapeutic target. To thoroughly investigate periostin expression, we decided to evaluate its presence at the molecular, tissue, and systemic levels by assessing mRNA, tissue protein, and serum levels. By combining data from molecular analyses and histopathological assessments, we seek to elucidate periostin’s multi-dimensional role in the pathogenesis and progression of these conditions, contributing to the broader understanding of its multifaceted functions in human health and disease.

## 2. Materials and Methods

### 2.1. Study Population and Inclusion Criteria

This was a single-center observational study conducted in the Otorhinolaryngology Department of the University Hospital of Patras between September 2021 and January 2024. Tissue sampling and clinical data collection was approved by the Research and Ethics Committee of the University Hospital of Patras (number 352/9 September 2021) after submission and review of the study protocol. All subjects have provided written informed consent before their recruitment concerning acquisition and processing of personal data and use of excised tissues for the purposes of the study. All study procedures and associated research were adherent to the principles of the Declaration of Helsinki.

Sixty-six participants (18 patients with inverted papillomas, 10 with mucoceles, 10 with choanopolyps, 16 with retention cysts and 12 controls) were prospectively recruited for this study. Inclusion criteria for enrollment in the patient group were patients over the age of 18 with a confirmed histopathologic diagnosis of the above-mentioned pathologies (inverted papilloma, mucocele, choanopolyp, cyst) and scheduled to undergo functional endoscopic sinus surgery (FESS). Standard computed tomography (CT) and endoscopy were performed preoperatively for all patients.

CRSsNP, allergic fungal rhinosinusitis, and CRSwNP were considered exclusion criteria. None of the patients received intranasal, oral and/or intramuscular corticosteroids within the 4 weeks before surgery. For female participants, pregnancy and/or lactation was guarded as exclusion criteria. Participants were selected randomly solely based on consecutive appointments for scheduled surgery. The control group consisted of patients who underwent septoplasty for nasal septum deviation without any other nasal pathology.

### 2.2. Biological Samples Acquisition

Tissue samples of nasal polyp were collected from 66 patients, 18 with inverted papillomas, 10 with mucoceles, 10 with choanopolyps and 16 with cysts, undergoing FESS, while control specimens were obtained from 12 patients with nasal septum deviation. A section of the excised tissue was sent for histologic examination, while the remainder was stored at −80 °C until processed.

Whole blood samples were collected preoperatively upon patient admission. The samples were allowed to clot at room temperature for 10–20 min and then centrifuged at 3000 rpm for 20 min for separation of serum. The latter was stored at −80 °C and thawed immediately before use.

### 2.3. Real Time Polymerase Chain Reaction (qRT-PCR)

The tissue samples were frozen at −80 °C until total RNA was extracted using the NucleoSpin RNA/protein kit (Macherey-Nagel, Duren, Germany) according to the manufacturer’s instructions. Homogenization of the samples was performed, and the instructions were followed as indicated until elution of total RNA. Total RNA was measured with a Multiskan Sky High Microplate Spectrophotometer (Thermo Fischer, Waltham, MA, USA). Measurements were performed at 230 nm and 280 nm to ensure RNA purity. The estimated ideal absorbance ratio at 260 nm (DNA) versus 280 nm (proteins) was 1.8. The quality of RNA in all specimens was also assessed with a 1.5% agarose gel in the presence of formaldehyde. A total of 5 µg of total RNA per sample was reverse transcribed to complementary DNA (cDNA) using Superscript II reverse transcriptase (Invitrogen, Carlsbad, CA, USA). cDNA was synthesized using random nucleotide hexamers as primers. qPCR reactions were performed using the KAPA SYBR FAST qPCR Kit (Kapa Biosystems, Wilmington, MA, USA), with 50 ng cDNA as a template. Reactions were set up in 96-well plates and performed on an MX3000P qPCR system (Agilent, Santa Clara, CA, USA). The Ct values were analyzed using the 2^−ΔΔCT^ method after normalization against GAPDH levels (20). The normalization procedure was performed using one of the control samples (sample C1). All reactions were performed in triplicates and the primers’ sequences are the following: GAPDH_F: GTCTCCTCTGACTTCAACAGCG, GAPDH_R: ACCACCCTGTTGCTGTAGCCAA, POSTN_F: TTGATGGAGTGCCTGTGGAA, POSTN_R: AACTTCCTCACGGGTGTGTC.

### 2.4. Western Blotting

Protein lysates were extracted using the NucleoSpin RNA/protein kit (Macherey-Nagel) according to the manufacturer’s instructions. Thirty µg of total protein extracts were separated by SDS-PAGE and transferred to Immobilon-P PVDF membranes (Millipore, MA, USA). Blocking was performed in 5% (*w*/*v*) non-fat dry milk in TBS/0.05% Tween 20. Incubation with primary antibodies was performed overnight at 4 °C. Incubation with goat anti-rabbit secondary HRP-conjugated antibody was performed for 2 h at room temperature. The band intensity measurements of each experiment were analyzed using the Image Lab software (Bio-Rad, Hercules, CA, USA, version 6.1). The protein levels were normalized against GAPDH levels in each separate set of experiments. The normalization procedure was performed using one of the control samples (sample C1). The antibodies used were the following: anti-GAPDH (#2118, Cell Signaling, Danvers, MA, USA) and anti-POSTN (#91771, Cell Signaling).

### 2.5. ELISA

Measurement of serum POSTN levels was performed using the Human Periostin/OSF2 ELISA Kit (EA100631, Origene, Rockville, MD, USA) according to the manufacturer’s instructions. Briefly, diluted serum was incubated for 2 h at room temperature. The detection antibody was incubated for 1 h at room temperature. Diluted streptavidin-HRP solution was incubated for 1 h at room temperature. Incubation of soluble TMB substrate for 30 min was followed by incubation with stop solution for 2 min. Absorbance at 450 nm was measured with a Multiskan Sky High Microplate Spectrophotometer (Thermo Fischer). Calculation of serum POSTN levels (ng/mL) was performed using a standard curve by fitting the results of the protein standard data to a model equation.

### 2.6. Statistical Analysis

Statistical analysis was performed using the GraphPadPrism 8 software package. The unpaired parametric Student’s *t*-test was applied and measurement data for all samples were expressed as mean ± standard deviation (SD). Prior to conducting the unpaired Student’s *t*-test, the dataset was visually inspected for approximate normality using histograms, and the distribution appeared to be approximately normal. A *p*-value < 0.05 (*) was considered statistically significant.

## 3. Results

Sixty-six participants (18 patients with inverted papillomas, 10 with mucoceles, 10 with choanopolyps, 16 with retention cysts and 12 controls) were prospectively recruited for this study. Clinical and demographic data of the patient and control groups are summarized in Table 1 and Table 2. The demographic data, including age and gender distribution, were comparable across the groups. The control group had an average age of 45.2 years, while the inverted papillomas group averaged 61.8 years. The choanopolyps and mucocele groups had average ages of 58.7 and 40.9 years, respectively. Notably, the cysts group had an average age of 40.2 years. (Table 1 and Table 2).

### 3.1. Expression Profile of POSTN mRNA, Protein and Serum Levels in Inverted Papillomas

**Figure 1:**
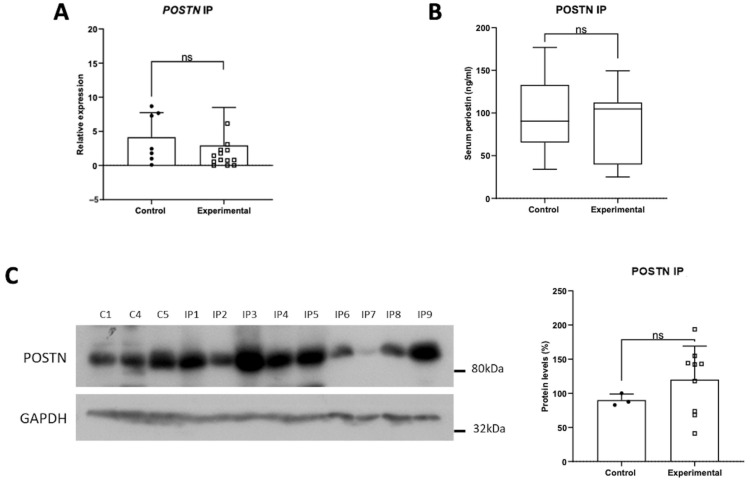
Expression of POSTN in inverted papillomas patients, as indicated by (**A**) the mRNA levels, (**B**) serum levels, and (**C**) protein levels of POSTN. Data are compared using unpaired Student’s *t*-test and are presented as means ± SD. *p*-values are indicated with ns, non-significant.

The expression profile of POSTN mRNA showed no statistically significant alteration in the inverted papillomas tissue samples compared to the control group (Figure 1A). No statistically significant alterations were observed in POSTN serum levels or tissue protein levels (Figure 1B,C), indicating that POSTN may not be involved in the pathogenesis or progression of inverted papillomas.

### 3.2. Tissue POSTN mRNA and Protein Expression Profile and Comparison with Serum Levels in Choanopolyps

**Figure 2:**
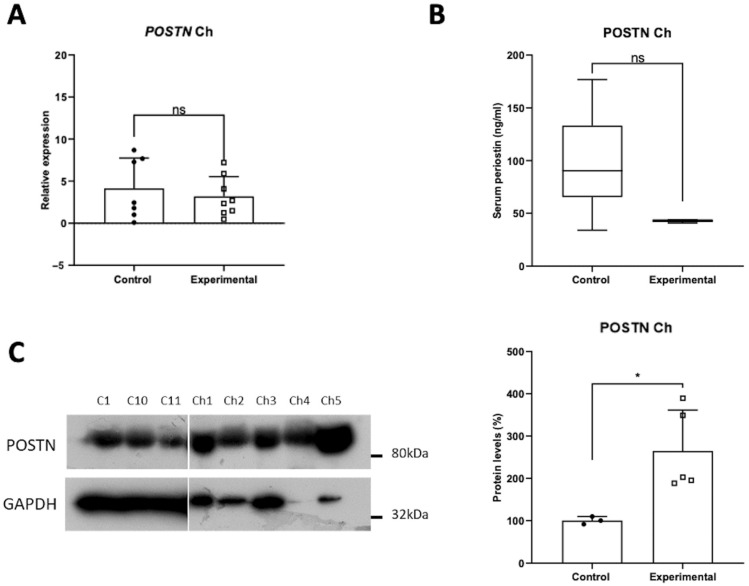
Expression of POSTN in choanopolyps patients, as indicated by (**A**) the mRNA levels, (**B**) serum levels, and (**C**) protein levels of POSTN. Data are compared using unpaired Student’s *t*-test and are presented as means ± SD. *p*-values are indicated with * *p* < 0.05; ns, non-significant.

POSTN mRNA levels showed no statistically significant alteration in the choanopolyps tissues compared to the control (Figure 2A). The serum protein levels of POSTN, as measured by ELISA, also showed no statistically significant alterations (Figure 2B). However, western blot analysis showed a statistically significant increased expression of POSTN protein levels in choanopolyps tissues compared to the control group with a more than two-fold increase (Figure 2C).

### 3.3. Tissue POSTN mRNA and Protein Expression Profile and Serum Levels in Mucoceles

**Figure 3:**
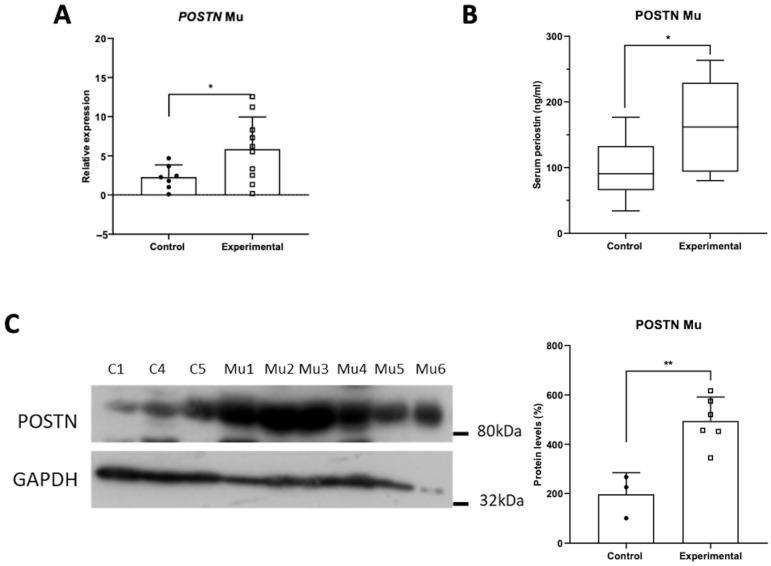
Expression of POSTN in mucoceles patients, as indicated by (**A**) the mRNA levels, (**B**) serum levels, and (**C**) protein levels of POSTN. Data are compared using unpaired Student’s *t*-test and are presented as means ± SD. *p*-values are indicated with * *p* < 0.05; ** *p* < 0.01.

The results for mucoceles indicated a clear upregulation of POSTN expression. Quantitative PCR revealed a statistically significant increase in POSTN mRNA levels in mucoceles compared to control samples (Figure 3A). Moreover, serum POSTN levels were also found to be elevated in patients with mucoceles (Figure 3B). This upregulation was further corroborated by western blot analysis, which demonstrated a two-fold increase in POSTN protein levels within mucoceles tissues (Figure 3C). These findings imply that periostin may play an important role in the pathophysiology of mucoceles, possibly relating to its involvement in tissue remodeling and repair mechanisms.

### 3.4. Expression Profile of POSTN mRNA, Protein and Serum Levels in Cysts

**Figure 4:**
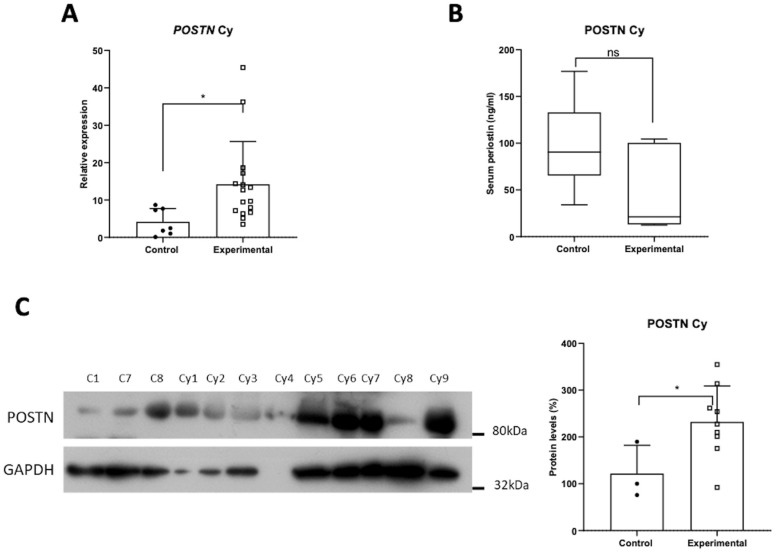
Expression of POSTN in cysts patients, as indicated by (**A**) the mRNA levels, (**B**) serum levels, and (**C**) protein levels of POSTN. Data are compared using unpaired Student’s *t*-test and are presented as means ± SD. *p*-values are indicated with * *p* < 0.05; ns, non-significant.

POSTN mRNA and protein levels of POSTN were upregulated in the cysts’ tissues compared to the control group, indicating POSTN’s possible involvement in cyst pathogenesis or progression (Figure 4A,C). The serum levels of POSTN, on the other hand, showed no significant alteration (Figure 4B).

## 4. Discussion

This study aimed to explore the expression of periostin in benign lesions of the nose and paranasal sinuses, focusing on a variety of different pathologies including mucoceles, inverted papillomas, choanopolyps, and cysts. Our findings revealed distinct periostin expression profiles across these conditions, suggesting periostin’s varied roles in their respective pathophysiology. The expression of periostin was not significantly altered in inverted papillomas patients compared to controls, indicating a possible limited role of periostin in this condition’s pathogenesis. In contrast, choanopolyps’, mucoceles’, and retention cysts’ tissue samples exhibited elevated levels of periostin, pointing to its possible involvement in these lesions.

To gain a comprehensive understanding of periostin expression, we elected to analyze its presence across molecular, tissue, and systemic levels by measuring mRNA, tissue protein levels, and serum levels. This multi-level approach facilitated the tracing of the entire periostin expression pathway, thereby providing insights into the variations observed at each stage. Such an integrative approach not only enhances our understanding of periostin’s role in these conditions but also enables the identification of the most reliable and practical methods for its evaluation.

Our findings indicate that mRNA and tissue protein levels are the most reliable indicators of periostin expression, as they directly reflect periostin’s activity at a local level where these lesions develop. In contrast, serum periostin levels, while valuable in understanding systemic changes, are more prone to variability possibly due to other comorbidities such as asthma, allergic rhinitis and atopic dermatitis [11,12,13,14]. These confounding factors may limit the applicability of serum periostin levels as standalone biomarkers, emphasizing the need for caution when interpreting such measurements.

### 4.1. POSTN and Sinonasal Inverted Papilloma (SNIP)

Inverted Papilloma is one of the most common neoplastic conditions of the nose and paranasal sinuses. Although benign in nature, it has a high recurrence rate (up to 75%), and has the tendency to invade neighboring structures causing bone erosion and it rarely undergoes malignant transformation (5–15%) [15]. Thus, early diagnosis, meticulous operational excision and thorough post operational follow-ups are crucial for optimal management. SNIP often presents as unilateral polypoid-like mass on endoscopy, thereby justifying its inclusion for comparative purposes in our study. Unlike the other lesions examined in this study, SNIP pathogenesis involves tumor-associated pathways rather than fibrosis or collagen deposition, as evidenced by abnormal expression of gelsolin, cathepsin S, PCBP2, IRF-1, and p53 [16]. This distinction likely explains why periostin was not significantly elevated in IP tissue or serum in our findings. Importantly, periostin’s non-elevation in SNIP highlights its potential as a biomarker to differentiate IP from unilateral polyps or CRSwNPs, adding value to its diagnostic utility.

### 4.2. POSTN and Antrochoanal Polyps

Antrochoanal polyps (ACPs) are benign mucosal lesions usually originating from the oedematous mucosa of the maxillary sinus in contrast to inflammatory nasal polyps, which originate from the ethmoid cells [17]. The main difference between NPs and ACPs is that the latter are typically unilateral with only very few cases of bilateral APCs reported in the literature [18]. Macroscopically, these lesions consist of a cystic part inside the sinus cavity, which grows through the main or accessory ostium into a solid polypoid part in the nasal cavity extending posteriorly to the choana and nasopharynx [19]. The histological differences between ACP and bilateral ethmoidal polyps are evident and can be characterized by an overall reduction in inflammatory infiltrate and eosinophil infiltrates and an increase in the number of neutrophils and macrophages [20].

Despite the significant amount of research conducted on the pathophysiology of nasal polyps in recent years, there has been a paucity of evidence exploring the pathophysiologic mechanisms of ACPs. In the largest study to date, of 200 cases of ACP, Frosini et al. suggest that the development of ACP may be primarily due to anatomical causes. In-creased pressure in the maxillary sinus caused by an inflammatory anatomical change at the level of the ostiomeatal complex/middle meatus in a patient with a pre-existing silent antral cyst, may subsequently be forced to herniate outwards through the ostium [18]. ACPs display a T1-dominant endotype immune response—with the presence of macrophages and CD8+ T cells and increased tissue remodeling, fibrin deposition and oedema [21]. Furthermore, the T1-dominant endotype is also associated with the higher levels of IFN-γ, IL-6, IL-8 and IL-10 in ACP compared to normal mucosa, eosinophilic and non-eosinophilic CRSwNP [20]. As observed by Bachert and Van Cawenberger, elevated IL-6 and neutrophilic activity further align ACPs with acute infection-like responses, rather than chronic bilateral polyposis [22]. ACP also had higher levels of neutrophil infiltration and expression of myeloperoxidase (MPO), interleukin (IL)-8 and interferon (IFN)-γ [23]. Our findings of elevated periostin in ACPs, despite its association with Th2 inflammation in NPs, point out the role of periostin in fibrotic remodeling and wound healing. Periostin is not exclusive to Th2 responses; it is also associated with structural changes common to ACPs. In the oral mucosa, periostin upregulates ECM components such as fibronectin and collagen via the FAK/JNK signaling pathway and is increased in connective tissue post-injury [24]. It is therefore possible that, similar to its role in oral mucosa, periostin may contribute to tissue remodeling in nasal and paranasal mucosa. This dual role could explain the elevation of periostin in ACPs, which implies an interplay between inflammatory and remodeling pathways in nasal polyp subtypes.

### 4.3. POSTN and Mucoceles

Mucoceles are benign cyst-like formations of the paranasal sinuses lined by the mucoperiosteum of the sinus cavity. Although primary mucoceles can occur, most of them appear to be secondary following trauma or surgery. They form mainly due to obstruction of a sinus cavity that leads to disruption of the normal mucociliary drainage.

The chronic inflammation within the mucocele results in the release of pro-inflammatory cytokines, collagenases and prostaglandins which cause osteoclastic bone resorption. IL-1, IL-6 and TNF-a have been detected in mucoceles [25]. They have the tendency to progressively expand causing remodeling and bone erosion in surrounding structures like the orbit or the intracranial cavity [26]. In our study, both periostin and the POSTN gene were found significantly upregulated in mucoceles’ tissue compared to controls while POSTN serum levels were also elevated. The progression of mucoceles and possibly the bone repair response triggered by them, that involves periostin as a key modulator of regeneration processes, may be the cause behind these findings.

### 4.4. POSTN and Paranasal Sinus Retention Cysts

Our study demonstrates a significant upregulation of POSTN mRNA and protein levels in cyst tissues compared to the control group, suggesting a potential role for periostin in the pathogenesis or progression of cysts. Serum levels of POSTN showed no significant alternation, indicating that the increased expression is localized to the cyst tissue itself. While research specifically on POSTN in nasal cysts is scarce, insights from studies on autosomal dominant polycystic kidney disease (ADPKD) provide valuable context for understanding its potential functions in cyst formation and progression. In ADPKD, POSTN is highly expressed in cyst-lining epithelial cells and accumulates within the matrix surrounding the cysts [27]. Further research has demonstrated that periostin overexpression in collecting ducts accelerates renal cyst growth and fibrosis [28].

Our findings, although in a different organ system, mirror this pattern, with periostin accumulation observed in tissues rather than in the periphery, thereby suggesting a localized role for periostin with its effects primarily confined to the cyst microenvironment, maintaining cyst structure and potentially promoting growth. In ADPKD, periostin interacts with αVβ3 and αVβ5 integrins, stimulating integrin-linked kinase to promote cell proliferation [29]. Similar interactions between periostin and integrins have been observed in airway epithelial cells, where they play a role in tissue remodeling in both CRSwNP and asthma [30]. While our study did not specifically investigate these interactions, the similar localization pattern suggests a potentially conserved mechanism of periostin action across different epithelial tissues, highlighting its importance in various pathophysiological processes.

### 4.5. Limitations

This study has several limitations that should be considered when interpreting its results. These include the relatively small sample size across groups and the single-center design, which may limit statistical power and generalizability of our findings to broader populations. Additionally, the selection of participants based on consecutive appointments for scheduled surgery may have introduced some selection bias. Despite the efforts made to account for localized perinasal factors, the study did not sufficiently address the potential impact of systemic comorbidities and environmental influences on serum periostin levels. These include Th2-mediated pathologies such as asthma, allergic rhinitis, and atopic dermatitis, as well as other comorbidities such as cardiac, pulmonary, dermatological, and neoplastic diseases [6]. It is possible that the influence of these systemic conditions may impact the interpretation of serum periostin levels

## 5. Conclusions

In conclusion, this study underscores the differential expression of periostin in benign lesions of the nose and paranasal sinuses, providing valuable insights into their pathophysiology and opening avenues for potential therapeutic exploration. Our findings suggest that periostin may play a role in the pathogenesis of different lesions, with mRNA and tissue protein levels emerging as the most reliable indicators of its local activity.

Future research should focus on optimizing the diagnostic and prognostic value of periostin, aiming to balance reliability with cost effectiveness to develop practical and accessible tools for clinical use. Ultimately, further investigation into periostin’s roles could pave the way for its application as both a biomarker and a therapeutic target in managing benign nasal and paranasal sinus conditions.

## Figures and Tables

**Table 1:** Patient group’s clinical and demographic data.

Patient	Gender	Age	Patient	Gender	Age
IP1	F	76	Mu1	M	36
IP2	M	45	Mu2	M	27
IP3	F	78	Mu3	M	83
IP4	M	48	Mu4	M	19
IP5	M	67	Mu5	M	39
IP6	F	72	Mu6	M	42
IP7	M	64	Mu7	M	49
IP8	F	65	Mu8	M	41
IP9	M	59	Mu9	M	56
IP10	M	44	Mu10	M	17
IP11	M	49	Cy1	M	36
IP12	F	57	Cy2	M	43
IP13	F	86	Cy3	M	45
IP14	M	73	Cy4	M	33
IP15	M	74	Cy5	F	56
IP16	F	46	Cy6	F	37
IP17	M	55	Cy7	F	41
IP18	F	54	Cy8	M	42
Ch1	M	58	Cy9	F	29
Ch2	M	59	Cy10	M	38
Ch3	F	67	Cy11	F	41
Ch4	F	13	Cy12	F	42
Ch5	F	78	Cy13	M	29
Ch6	M	71	Cy14	M	36
Ch7	F	69	Cy15	M	37
Ch8	F	61	Cy16	F	59
Ch9	M	56			
Ch10	F	55			

**Table 2:** Patient and control groups’ statistical data.

Controls					
Age					
Average (years)					
45.2					
(29.2–61.1)					
Gender					
			Percentage (%)		
	Male	8	66.7		
	Female	4	33.3		
**Inverted Papillomas (IP)**			**Mucoceles (Mu)**		
Age			Age		
		Percentage (%)			Percentage (%)
<40	-	-	<40	5	50.0
41–60	9	50.0	41–60	4	40.0
>61	9	50.0	>61	1	10.0
Gender			Gender		
Male	10	55.6	Male	10	100
Female	8	44.4	Female	-	-
**Choanopolyps (Ch)**			**Retention Cysts (Cy)**		
Gender			Gender		
		Percentage (%)			Percentage (%)
<40	1	10.0	<40	8	50.0
41–60	4	40.0	41–60	8	50.0
>61	5	50.0	>61	-	-
Gender			Gender		
Male	4	40.0	Male	9	56.2
Female	6	60.0	Female	7	43.8

## Data Availability

Data supporting this article can be made available by the authors upon request.
